# A Public Platform for the Verification of the Phenotypic Effect of Candidate Genes for Resistance to Aflatoxin Accumulation and *Aspergillus flavus* Infection in Maize

**DOI:** 10.3390/toxins3070754

**Published:** 2011-06-24

**Authors:** Marilyn L. Warburton, William Paul Williams, Leigh Hawkins, Susan Bridges, Cathy Gresham, Jonathan Harper, Seval Ozkan, J. Erik Mylroie, Xueyan Shan

**Affiliations:** 1 Corn Host Plant Resistance Research Unit, U.S. Department of Agriculture-Agricultural Research Service, MS 39762, USA; Email: Paul.Williams@ars.usda.gov (W.P.W.); Leigh.Hawkins@ars.usda.gov (L.H.); 2 Department of Computer Science and Engineering, Mississippi State University, MS 39762, USA; Email: bridges@cse.msstate.edu (S.B.); gresham@cse.msstate.edu (C.G.); jwh376@msstate.edu (J.H.); 3 Department of Biochemistry, Molecular Biology, Entomology, and Plant Pathology, Mississippi State University, MS 39762, USA; Email: so35@pss.msstate.edu (S.O.); jem135@msstate.edu (J.E.M.); XShan@BCH.msstate.edu (X.S.)

**Keywords:** aflatoxin, *Aspergillus flavus*, host plant resistance, candidate gene validation

## Abstract

A public candidate gene testing pipeline for resistance to aflatoxin accumulation or *Aspergillus flavus* infection in maize is presented here. The pipeline consists of steps for identifying, testing, and verifying the association of selected maize gene sequences with resistance under field conditions. Resources include a database of genetic and protein sequences associated with the reduction in aflatoxin contamination from previous studies; eight diverse inbred maize lines for polymorphism identification within any maize gene sequence; four Quantitative Trait Loci (QTL) mapping populations and one association mapping panel, all phenotyped for aflatoxin accumulation resistance and associated phenotypes; and capacity for Insertion/Deletion (InDel) and SNP genotyping in the population(s) for mapping. To date, ten genes have been identified as possible candidate genes and put through the candidate gene testing pipeline, and results are presented here to demonstrate the utility of the pipeline.

## 1. Introduction

Aflatoxins are carcinogenic and toxic metabolites produced by the fungus *Aspergillus flavus* during infection of maize and other seed oil crops. Hot and dry climatic conditions favor *A. flavus* infection and aflatoxin production in maize, adding an economic burden to the farmers and a health risk to consumers. One of the most promising avenues to combat aflatoxin contamination is the development of resistant maize lines, and several natural sources of resistance that exhibit significantly reduced aflatoxin accumulation have been identified [1]. However, transfer of resistance into elite breeding lines has proven difficult due to the highly quantitative nature of the trait and the high genotype by environment interaction. Genetic markers linked to or within genes that increase resistance would aid in the development of resistant inbreds and hybrids via Marker Assisted Backcrossing (MAB), if such genes were found to have a large enough phenotypic effect on the trait to make MAB worthwhile. In many previous reports, candidate genes for resistance to *A. flavus* infection and aflatoxin production have been identified via Quantitative Trait Loci (QTL) mapping, genomics, or proteomics studies. Published QTL mapping studies have identified between 2 and 10 QTL per study, each with a small but measureable effect on the phenotype [1,2,3,4,5,6,7]. The use of genomics and proteomics tools has identified hundreds of gene and protein sequences that are differentially regulated in response to *A. flavus* infection between resistant and susceptible genotypes [8,9,10].

Although many candidate loci and gene sequences have now been identified, none have been reported in use to create improved breeding lines to date. There are several possible explanations why they have not been used. Few QTL have been identified with large phenotypic effects or that are consistent across multiple environments, and they have rarely been tested in more than one genetic background. The candidate genes identified via differential expression profiling of mRNA or protein have generally not been validated and cannot yet be confirmed to have a causal effect on the trait. In addition, hundreds of potentially up- or down-regulated genes have been identified, which is too many to begin to use in a practical breeding program. Even with the most advanced breeding technology available, there is no feasible method to use these large numbers of informative genes simultaneously for breeding purposes. The problem is confounded because resistance genes identified to date are usually found in genetic backgrounds that differ from US breeder’s elite materials. Breeders generally will not invest in these genes without more guarantee of success. The most conclusive independent validation of these genes would require the production and field testing of near isogenic or transgenic lines. Because of the expense and time required to generate these lines, only the most promising and well characterized candidate genes should be tested with them. 

Here, we present a candidate gene testing pipeline consisting of a database for choosing candidate genes, a panel of maize lines for identifying polymorphisms within the genes, and mapping populations for testing the phenotypic effect of each gene. Genes which appear to significantly reduce aflatoxin accumulation or fungal biomass can then be used to create Near Isogenic Lines (NILs) for final validation of gene effect. Ten candidate genes have been taken through part or all of the steps in the pipeline. The objectives of this study were to follow these 10 genes through the candidate gene testing pipeline and ensure that the steps are all efficient, necessary, and yield conclusive results. These results are presented here.

## 2. Materials and Methods

**Figure 1:**
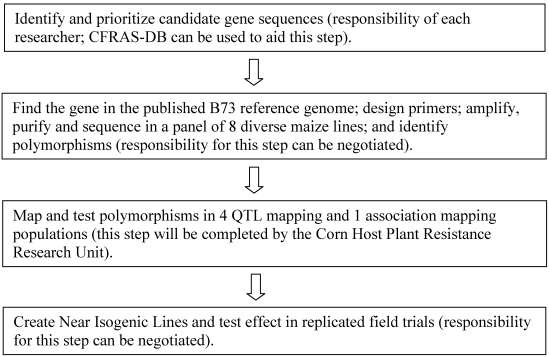
Diagram of the steps in the candidate gene testing pipeline. Researchers interested in submitting gene sequences to be analyzed in the pipeline can contact the corresponding author of this article at the USDA/ARS Corn Host Plant Resistance Research Unit.

The overall flow of the candidate gene testing pipeline can be found in Figure 1. Resources associated with the pipeline include the following.

**Figure 2:**
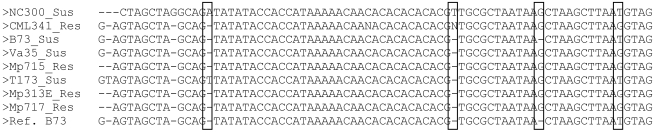
Example of an alignment of the 8 diverse inbred lines and the B73 reference (published genotype) used to find polymorphisms within one amplicon of the p450 candidate gene, and compared to the published reference B73 genome sequence. Boxes identify possible SNP or InDel polymorphisms between the lines that could be tested in the QTL and association mapping populations.

1. The Corn Fungal Resistance Associated Sequences Database (CFRAS-DB) of DNA and protein sequences identified in past resistance studies [9]. This database is implemented in MySQL 5.1.31-community version and contains fifteen core tables with data from both local and external sources. Data sources include maize microarray datasets, maize proteomics datasets, QTL data, and SNP data. Data have unique identifiers that relate one table to another, using DNA or protein sequence IDs or genomic location [11], which allow the user to combine data from more than one table, and thus find gene and protein sequences that may be good candidates based on multiple studies. 

2. A diverse panel of 8 inbred lines for SNP and InDel (Insertion/Deletion polymorphisms) identification within any given maize gene sequence. The lines include four resistant lines (Mp313E, Mp715, Mp717, and CML341) and four diverse susceptible lines (B73, NC300, T173, and Va35), chosen because they are well characterized (in the case of B73) or frequently used as breeding lines in the southern US. In addition, because they are from unrelated germplasm, there is a higher probability of finding polymorphisms between the lines within each candidate sequence. These lines are currently being re-sequenced to identify polymorphisms within any selected candidate gene sequence. Genes can also be quickly sequenced one at a time in the panel as part of the pipeline (Figure 2). Following sequencing of the 8 lines, a BioPerl script created in our laboratory and available upon request from the corresponding author is used to combine forward and reverse sequencing runs of the same genotype into a consensus sequence. Mismatches at any base in the forward and reverse sequences are regarded as missing data unless one of them is the same as the B73 reference sequence in which case that is what is used at that position in the sequence. Consensus sequences are then trimmed up and downstream of the primers used to amplify the sequence in each line, and compared to identify SNPs or InDel polymorphisms between genotypes. Consensus sequences with more than 5% missing bases are re-sequenced or removed from the study.

InDels identified within each gene sequence are used to design a size-based assay that can be run via PCR and visualized on an agarose or polyacrylamide gel, and SNPs are used to create a SNP assay that can be run on a fluorescence-based plate reader. In our laboratory, size based polymorphism assays are designed by finding primer pairs within the candidate sequence using Primer 3 software version 0.40 (http://frodo.wi.mit.edu/primer3/). These are ordered from Integrated DNA Technologies (Skokie, IL, USA), and tested for amplification on the 8 lines from the diverse panel, above, followed by electrophoresis on agarose or polyacrylamide gels. KASPAR assays for SNP based polymorphisms are designed from the candidate SNP sequences identified in the 8 diverse lines by KBiosciences (Hoddesdon Herts, UK) and run on a BMG LabTech FLUOstar Model 403 Fluorometer (Cary, NC, USA).

3. Four QTL mapping populations and one association mapping panel. The QTL mapping populations include F_2:3_ families of the crosses between Mp313E × Va35 (MpVa), Mp313E × B73 (MpB), Mp715 × T173 (MpT), and Mp717 × NC300 (MpNC) (resistant parents are listed first in each cross). These populations have each been genotyped with over 100 Simple Sequence Repeat (SSR) and Restriction Fragment Length Polymorphism (RFLP) markers. The association mapping panel contains 300 inbred lines, including all aflatoxin/*A. flavus* resistance sources known at the time of panel formation, and many other lines chosen to represent the diversity present in the US and global maize gene pool. All lines in the association mapping panel were test-crossed to Va35, a Southern adapted US inbred with low levels of resistance to aflatoxin/*A. flavus* accumulation. All lines are currently being re-sequenced and this data will be available soon. 

All five populations have been phenotyped over multiple years, locations, and two to three replications for aflatoxin accumulation resistance and associated phenotypes, including fungal biomass calculated via qPCR according to Mideros *et al*. [12], ear rot ratings, husk coverage, and earworm damage. Due to the high cost, fungal biomass via qPCR was measured in only one year for three locations and both years in only one location for the association panel. 

**Figure 3:**
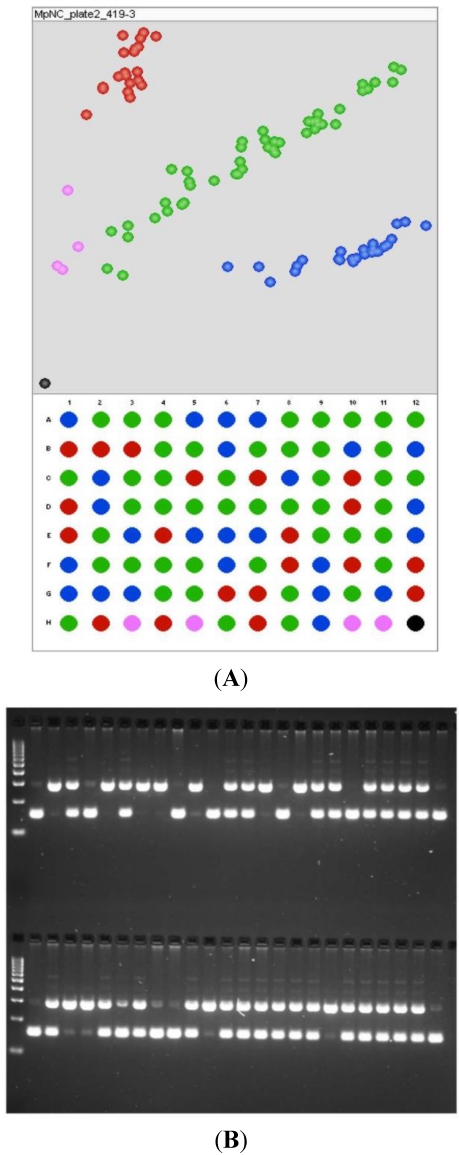
Example of genotyping available for polymorphisms to be tested in the QTL mapping population. The markers shown here segregate in the expected 1:2:1 pattern for F_2_ individuals. (**A**) SNP genotyping showing the genotype of the parents (well A1 and B1), F_1_ (well A2), and no-template control (well H12, the black spot), as well as four ambiguous (and thus missing) data points (in pink); (**B**) InDel genotyping showing the parents (two lanes following the molecular weight standard on the top tier of the InDel gel image, as read left to right) and F_1_ (third lane).

4. Genotyping capability to allow each InDel and SNP identified in the pipeline to be characterized in the population(s) (Figure 3). Following the data generation, analyses in the QTL mapping populations proceeds as follows: linkage mapping is run using the JoinMap mapping software (version 4) [13] using the Maximum Likelihood (ML) mapping function, and compared to published map orders in the Maize Genetics and Genomics Database [11]. QTL effects are measured using the Composite Interval Mapping function of QTL cartographer version 2.5 [14]. Association analysis is performed using TASSEL version 3.0 [15], using the General Linear Model (GLM) and the kinship matrix between all lines in the panel. The Mixed Linear Model (MLM) can also be used when relationships between all lines in the panel are calculated from SNPs generated from the re-sequencing for these 300 lines. GLM and MLM will indicate if each candidate gene has an effect on the phenotype in the population.

Ten candidate genes were chosen to be run through the pipeline based on queries of the CFRAS database including genes or proteins significantly up- or down- regulated in resistant lines following infection with *A. flavus*, and co-localization with previously reported QTLs; or were chosen from the published literature (Table 1). Candidate gene sequences were taken from the published maize genome sequence database [16], NCBI sequence database [17] or UniProtKB protein sequence database [18]. All steps in the pipeline except association mapping were tested on all ten genes. Association mapping was only tested in one gene, Photosytem II3. In this gene, five hundred forty bases were sequenced in all lines in the association mapping panel. Two hundred forty of these lines successfully yielded useable consensus sequences and were used in the association test. 

**Table 1:** Information on the 10 candidate genes included in this study including the identification number from the corresponding published database; the name or putative function of the gene, location on the chromosome according to the published maize genome sequence; and a published reference, when available. Sequences chosen on the basis of queries from the CFRAS-DB database are indicated, if no previous studies on resistance have been published for these candidate genes.

MaizeSequence, EST or UniProt ID	Gene Name	Chromosomal Location *	Reference
Q43257	Cytochrome P450	4:3260685	[19]
A2SZW8	1-Cys peroxiredoxin PER1	7:168,785,597	[8,20]
CF038389	Hypothetical protein	3:140291029	CFRAS-DB query
TC221535	Unk. homocysteine S-methyltransferase	3:117292752	CFRAS-DB query
TC230106	Hypothetical protein	4:155846482	CFRAS-DB query
TC237439	Hypothetical protein	4:85917427	CFRAS-DB query
AY241545.1	Glyoxalase I	10:4444027	[21]
DQ335768.1	Lipoxygenase 10	4:239236528	[8,20]
GRMZM2G007555	Heat Shock Protein (22 kD)	1:167255241	[22]
AW424439	Photosytem II3 protein, chloroplast precursor	4: 27096658	[22]

* Location is read as chromosome number before the colon and base pair number after the colon.

## 3. Results and Discussion

**Figure 4:**
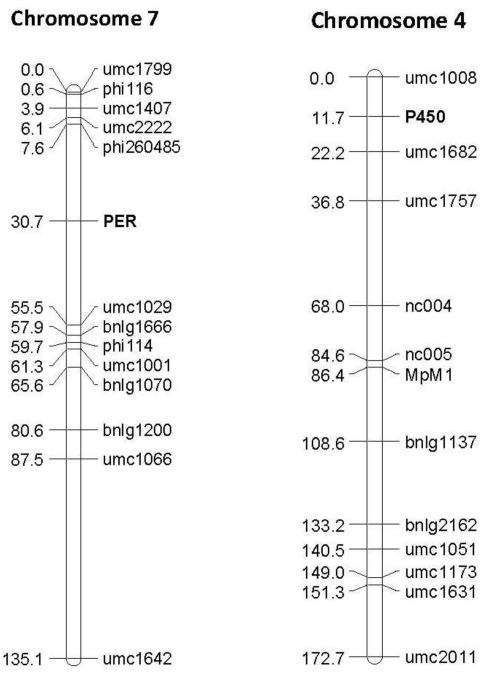
Examples of linkage maps generated in the F_2:3_ segregating population Mp715 × T173, showing the location of the gene based markers 1-Cys peroxiredoxin PER1 (PER) and Cytochrome P450 (P450), and previously mapped SSR markers. Both markers map to the expected location based on the location found by BLAST against these sequences in the B73 reference genome. The numbers to the left of the chromosomes are the cM distances calculated between markers by the JoinMap linkage mapping program.

One to six size-based or Single Nucleotide polymorphisms were identified within the sequences of all ten candidate genes over the 8 diverse inbred lines, and were used to generate assays to map the genes in the four QTL mapping populations. Size assays (for the genes photosytem II3, P450 and PER1) were amplified and separated on agarose gels for mapping in the QTL populations for which these InDels segregated; SNP assays (for all other genes) were visualized with the KASPAR (KBioScience, Hoddesdon Herts, UK) SNP detection system. This was found to be the most economical method for running a smaller number of SNPs on many lines. The polymorphisms for all ten genes were mapped to the expected places on the chromosomes in all cases using JoinMap (Table 2) and an example of two of the maps generated with these markers is shown in Figure 4. 

**Table 2:** Effect of candidate gene polymorphisms on the phenotype measured in QTL mapping populations. Quantitative trait loci (QTL) associated with resistance to *A. flavus*/aflatoxin have been identified in four bi-parental populations: Mp313E × Va35 (MpVa), Mp313E × B73 (MpB), Mp715 × T173 (MpT), and Mp717 × NC300 (MpNC).

MaizeSequence, EST or UniProt ID	Gene Name	QTL Population in Which Polymorphic	LOD at the Peak of Significant QTL Effect	Potentially Useful Marker for Fine Mapping?
Q43257	Cytochrome P450	MpT, MpVa	MpVa = 2.5	yes
A2SZW8	1-Cys peroxiredoxin PER1	MpT, MpVa, MpNC	none	no
CF038389	Hypothetical protein	MpNC	none	yes
TC221535	Unk. homocysteine S-methyltransferase	MpT	none	yes
TC230106	Hypothetical protein	MpB	MpB = 7.0	yes
TC237439	Hypothetical protein	MpNC, MpT	MpNC =2.5	yes
AY241545.1	Glyoxalase I	MpVa	none	no
DQ335768.1	Lipoxygenase 10	MpB, MpNc, MpVa	none	MpVa = yes, all others no
GRMZM2G007555	Heat Shock Protein (22 kD)	MpVa	none	no
AW424439	Photosytem II3 protein, chloroplast precursor	MpT, MpVa, MpB,	MpB = 2.5, MpVa = 2.4, MpB = 2.4	yes

**Figure 5:**
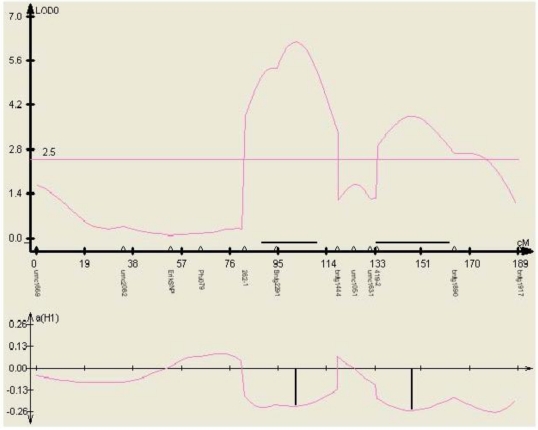
Phenotypic effect of the SNP marker 262-1 identified within gene TC230106, in three environments (mean aflatoxin levels measured at MSU in 2000, 2001, and 2002) in the mapping population Mp313E × B73, as calculated by QTL cartographer.

The phenotypic effect of each of the polymorphisms was estimated in the same mapping populations via QTL analysis. Polymorphisms in genetic sequences that explain a significant level of the phenotypic variation of one or more populations were found in four of the ten candidate gene sequences (Table 2, one example shown in Figure 5). Three of the genes with QTLs verified via linkage mapping (Q43257, TC237439, and AW424439) show new QTL that have not been reported in previous QTL mapping studies. One more (TC230106) falls within previously reported QTL, and may be the gene responsible for this QTL effect [2]. Finally, two genes (CF038389 and TC221535) did not appear to be associated with a phenotypic effect, but mapped close to and helped to further delineate the borders of previously reported QTL [5,6]. Such fine- mapping of QTL into smaller chromosomal fragments increases the value of the QTL in marker assisted selection by reducing the potential for linkage drag. 

Association mapping has been completed for only one gene to date (AW424439, Photosystem II3, from chromosome 4). Fifty-eight SNPs were found between these 240 lines, 40 of which had a minor allele frequency (MAF) of greater than 5%. A MAF of less than 5% does not lead to useful data, as this frequency is too low to have any statistical power in an association test due to small sampling size of one of the haplotype classes. In addition, there were 27 Indels (that ranged from 1 to 59 bp), of which 11 of these (all shorter than 2 bp) had a MAF of greater than 5%. There was also one SSR (with a GCG repeat) with various alleles (some very rare) in the middle of the amplified region. Such diversity makes it quite difficult to align the amplified regions and find polymorphisms, but this was done with the BioPerl script written expressly to reduce some of the complexity by aligning the forward and reverse sequences of each genotype and trimming sequences from up- and down-stream of the primer sites. Sequences with mismatched sites in the forward and reverse reads were not used. ClustalW (http://www.ebi.ac.uk/Tools/msa/clustalw2/), DNAMan (Lynnon Corporation, Pointe-Claire Canada) and TASSEL [15] were used for the alignment and polymorphism detection.

The 51 polymorphisms with MAF greater than 5% were run in TASSEL with the phenotypic data for the association mapping panel measured in Lubbock and College Station, TX, and Raymond and Starkville, MS, for 2010 only (2011 aflatoxin data are still being analyzed). No significant associations were found between any of the polymorphisms and aflatoxin levels in the panel in the site in Lubbock, TX. Three significant associations were found in data collected from College Station, TX (with probabilities of 9.27 × 10^−4^, 8.19 × 10^−4^, and 9.65 × 10^−4^). The latter two of these polymorphisms were completely linked, but the first was not. The two MS sites showed association between aflatoxin levels and one polymorphism (the same one) in both field sites, with probabilities of 3.86 × 10^−9^ in Raymond and 5.76 × 10^−37^ in Starkville. This polymorphism was not the same as those associated with aflatoxin in College Station, although it is linked to the College Station polymorphisms.

Although no significant association with a phenotypic effect in aflatoxin and *A. flavus* resistance according to linkage (QTL) mapping was found for six of the candidate genes tested here, it cannot be concluded that these candidate genes have no effect on the trait. It can only be concluded that they had no significant phenotypic effect in the mapping populations and the environments in which they were tested. It is possible that other polymorphisms in the same gene (alleles) could lead to improvements in resistance, but these polymorphisms were not present in any of the parents of any of our QTL mapping populations. This is a weakness of QTL mapping, and will be addressed by the concurrent use of an association panel of 300 diverse individuals (thus allowing the testing of many more possible polymorphisms simultaneously). On the other hand, many of the candidate genes found in the CFRAS database were identified in genomics or proteomics studies as related to *A. flavus* or aflatoxin resistance using the same parental lines of the QTL populations. Therefore, the likelihood that they will have large effects on resistance, but not be identified in these QTL populations, is small. In addition, the populations were grown under many different environmental conditions, and in some cases, in more than one genetic background. Thus, a lack of measureable phenotypic effect in the pipeline is actually quite suggestive that the gene being tested does not have a large effect on the trait.

## 4. Conclusions

The public candidate gene testing pipeline for aflatoxin accumulation or *A. flavus* resistance in maize presented here was used to quickly test the field-measured phenotypic effects of 10 candidate genes, and it was concluded that 6 of these had no effect on the traits (although two are good markers for fine mapping), and 4 had a small but measurable effect on the traits. The information presented here will allow these markers to be used for marker assisted improvement of this trait in maize. In addition, genes validated by this testing pipeline should be used in further (and more labor and cost intensive) studies, including the formation of transgenic or near isogenic lines to test for gene effect in multiple backgrounds, and seeking genes in a common pathway or network that will lead to a greater understanding of the interactions between maize, *A. flavus*, and the production of aflatoxin. Researchers working in the area of gene identification for reduction to aflatoxin accumulation or *A. flavus* infection in maize are encouraged to contact the corresponding author of this article if they wish to have their candidate genes run through the pipeline for independent validation of gene effect.
